# Network-Driven Reputation in Online Scientific Communities

**DOI:** 10.1371/journal.pone.0112022

**Published:** 2014-12-02

**Authors:** Hao Liao, Rui Xiao, Giulio Cimini, Matúš Medo

**Affiliations:** 1 Physics Department, University of Fribourg, Fribourg, Switzerland; 2 Institute for Complex Systems (ISC-CNR) and Department of Physics, “Sapienza” University of Rome, Rome, Italy; University Toulouse 1 Capitole, France

## Abstract

The ever-increasing quantity and complexity of scientific production have made it difficult for researchers to keep track of advances in their own fields. This, together with growing popularity of online scientific communities, calls for the development of effective information filtering tools. We propose here an algorithm which simultaneously computes reputation of users and fitness of papers in a bipartite network representing an online scientific community. Evaluation on artificially-generated data and real data from the Econophysics Forum is used to determine the method's best-performing variants. We show that when the input data is extended to a multilayer network including users, papers and authors and the algorithm is correspondingly modified, the resulting performance improves on multiple levels. In particular, top papers have higher citation count and top authors have higher *h*-index than top papers and top authors chosen by other algorithms. We finally show that our algorithm is robust against persistent authors (spammers) which makes the method readily applicable to the existing online scientific communities.

## Introduction

Science is not a monolithic movement, but rather a complex enterprise divided in a multitude of fields and subfields, many of which enjoy rapidly increasing levels of activity [1], [2]. Even sub-disciplines have grown so broad that individual researchers cannot follow all possibly relevant developments. Despite swift growth of online scientific communities (such as ResearchGate, Mendeley, Academia.edu, VIVO, and SciLink) [3] which facilitate social contacts and exchange of information, finding relevant papers and authors still remains a daunting task, especially in lively research fields.

At the same time, reliance of the modern society on computer-mediated transactions has provoked extensive research of reputation systems which compute reputation scores for individual entities and thus reduce the information asymmetry between the involved parties [4], [5]. What is perhaps more important than the immediately useful information is the proverbial shadow of the future—incentives for good behavior and penalties against offenses—generated by these systems [6], [7]. Reputation systems are now an organic part of most e-commerce web sites [8] and question & answer sites [9]. Complex networks [10] have provided a fruitful ground for research of reputation systems with PageRank [11], [12] and HITS [13] being the classical examples. In [14], the authors extended HITS by introducing authority score of content providers and apply the resulting EigenRumor algorithm to rank blogs. Building on BiHITS, a bipartite version of HITS [15], [16] presents a so-called QTR algorithm which has been developed for online communities. This algorithm co-determines item quality (which we refer to as fitness herein) and user reputation from a multilayer network which consists of a bipartite user-item network and a monopartite social network.

We propose here a reputation algorithm designed especially for online scientific communities where researchers share relevant papers. We first simplify the aforementioned QTR algorithm by neglecting the social network among users and thus obtain a new QR algorithm. This simplification reflects the fact that trust relationships are often not available and allows us to better study the algorithm's output with respect to the remaining parameters. We then devise a new QRC algorithm by introducing author credit which is however computed differently than in the previously-mentioned EigenRumor (note that we keep the previously used letter Q in the algorithm's name despite replacing the term *quality* with a more neutral term *fitness* in this paper). All three quantities—item fitness, user reputation, and author credit—represent reputation of three different kinds of entities that are present in the system.

**Figure 1:**
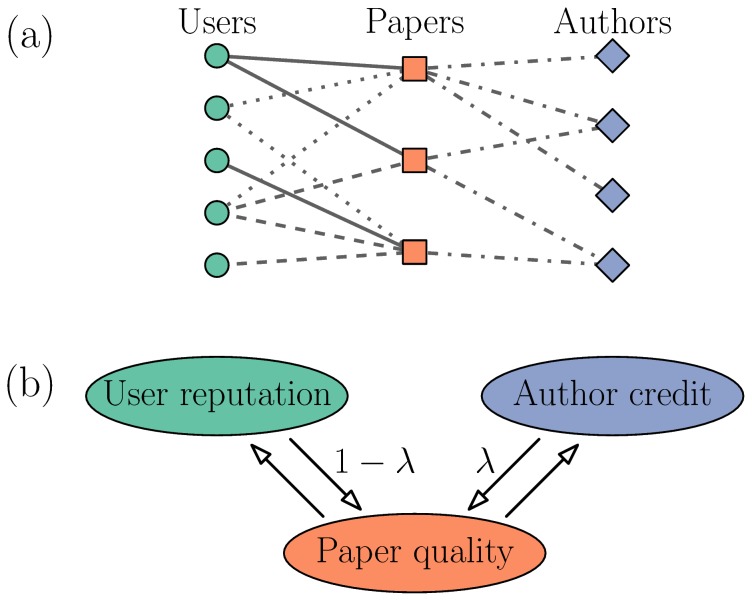
Schematic illustration of the data and the algorithm. (a) The input data can be represented by a multilayer network. Different line styles indicate different interactions: paper submission, download, and abstract view between users and papers, and authorship between papers and authors. (b) Score flows in the QRC algorithm.

Since author credit is co-determined from the same data as item fitness and user reputation, its introduction preserves an important advantage of QTR: reliance only on implicit ratings (represented by connections between users and items) which are easier to elicit than explicit ratings (scores given by users to papers) [8]. Similarly to various previous reputation algorithms [12], [17]–[19], the new algorithm can be effectively represented by score flows in a complex network. More precisely, the algorithm effectively acts on a multilayer network [20] consisting of two bipartite components: user-item and item-author network (see Figure 1 for an illustration). In the context of predicting future citation counts of papers, QRC represents an algorithm-focused alternative to machine-learning approaches [21], [22]. With respect to these and other works analyzing the patterns of scientific production [23], [24], the algorithm that we propose here differs in not relying on hard measures of research impact such as citation counts or journal impact factors (though we use some of these measures to validate the algorithm).

We first use artificial data produced by an agent-based model to evaluate and calibrate the basic version of the algorithm without author credit. The found best-performing algorithm variants are then used as a basis for the extended QRC algorithm with author credit. We apply the algorithm on real-world data and employ various metrics of research productivity to assess the best-ranked papers and authors and demonstrate that the new algorithm outperforms other state-of-the-art algorithms. Impact of the co-authorship network on author credit is discussed and two different scenarios are studied to show that the algorithm is robust with respect to persistent authors of low-fitness content.

## Methods

### Algorithms without author credit

An online community is assumed to consist of 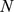 users and 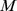 items (papers or other sort of scientific artifacts) which are labeled with Latin and Greek letters, respectively. The community is represented by a bipartite user-item network 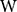 where a weighted link between user 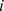 and item 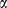 exists if user 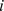 has interacted with item 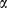. Link weight 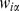 is decided by the type of interaction between the corresponding user-item pair and reflects the level of importance or intensity of the interaction. It is convenient to introduce an unweighted user-item network 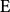 where 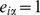 if 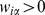 and 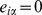 otherwise. The corresponding unweighted user and item degree are denoted as 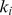 and 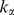, respectively.

We first introduce a bipartite variant of the classical HITS algorithm, biHITS, which assigns reputation values 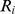 to user nodes and fitness values 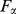 to item nodes. The algorithm's definitory equations are 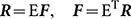
(1)where 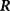 and 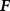 are *user reputation* and *item fitness* vector, respectively. Solution to this set of equations is usually found by iterations. Starting with 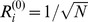 and 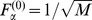, subsequent iterations are computed as
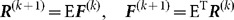
(2)and then normalized so that 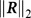 and 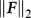 remain one. We stop the iterations when the sum of absolute changes of all vector elements in 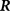 and 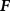 is less than 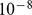. If 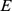 represents a connected graph, the solution is unique and independent of 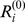 and 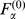 [13]. A weighted bipartite network can be incorporated in the algorithm by replacing the binary matrix 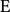 with the matrix of link weights 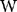.

We now simplify the QTR algorithm [16] by omitting *Trust* among the users—we refer it as the QR algorithm hence. Its definitory equations are 
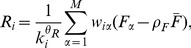
(3)

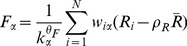
(4)where 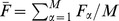 and 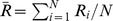 are the average fitness and reputation value, respectively. The algorithm is further specified by the choice of 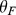, 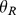, 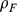, 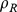 which all lie in the range 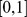. In particular, the two boundary choices of 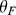 correspond to item fitness obtained by summing (when 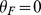) or averaging (when 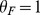) over reputation of all users connected with a particular item; the meaning of 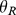 is analogous. By contrast, 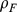 decides whether interactions with items of inferior fitness harm user reputation (when 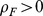) or not (when 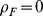); the meaning of 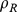 is analogous. Solution of Eqs. (3,4) can be again found iteratively. When 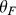, 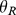, 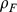, 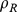 are all zero, QR differs from biHITS only in using the weighted matrix 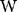 instead of 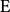.

### Algorithms with author credit

HITS-like algorithms that rely only on user feedback have two limitations. First, an item can only score highly after sufficient feedback has accumulated which can require substantial time in practice. Second, an item can attract the attention of users for fitness-unrelated reasons (by a witty or provoking title, for example) and the algorithms lack mechanisms to correct for this. EigenRumor algorithm (ER) responds to this by introducing scores for “information providers” [14] which we refer to as *author credit* here. While this algorithm originally includes only two sets of entities—blog entries and blog authors—it can be easily adapted to our case where users, papers, and authors are present.

The bipartite author-paper network can be represented by matrix 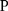 whose elements 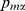 are 1 if author 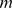 has (co)authored paper 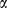 and 0 otherwise (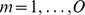 where 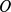 is the number of authors). Author and paper degree in this network are 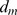 and 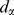, respectively. Denoting the vector of author credit values as 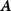, the equations of EigenRumor are an extension of Eq. (1), 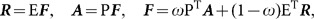
(5)where parameter 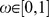 determines the relative contribution of authors and users to paper fitness. As noted in [14], matrices 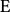 and 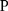 can be normalized to reduce the bias towards active users and authors. Normalization
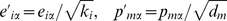
(6)is claimed to provide good results. Since the weighted user-paper interaction matrix 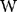 contains more information than 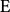, we use 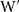 analogous to 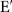 here.

To introduce author credit in the QR algorithm and thus obtain a new QRC algorithm (Quality-Reputation-Credit), we extend Eqs. (3,4) to the form 
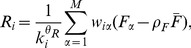
(7)

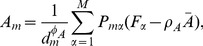
(8)

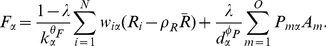
(9)


Parameter 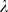 plays the same role as 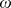 in EigenRumor. When 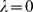, 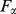 and 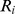 are the same as obtained by QR and author credit 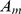 is computed simply as an additional set of scores. For any other value 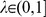, all three quantities depend on each other as illustrated by Fig. 1. Eqs. (7–9) can be again solved iteratively.

EigenRumor and QRC, albeit similar, show numerous differences. First, QRC uses three scores as opposed to two scores used by the original EigenRumor. Second, each summation term in QRC has its own normalization exponent (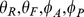) which decides how to aggregate over multiple user actions, authored papers, or co-authors. The absence of explicit normalization in EigenRumor Eqs. (5) is compensated by the eventual use of matrices 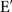 and 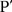 which makes ER's equations for 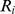 and 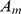 similar (up to a different value of exponent) to those of QRC. However, the ER's equation for 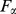 is based on 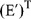 and 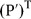 which implies terms 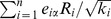 and 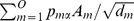 without counterparts in Eqs. (7–9).

### Model evaluation on artificial data

We now describe an agent-based system [25] which aims at producing data that can be analyzed by the benchmark QR algorithm. We aim to evaluate the algorithm's performance by comparing the true values of fitness and reputation with those produced by the algorithm.

In the agent-based system, each user 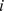 is endowed with intrinsic ability 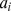 and activity level 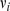, whereas each item 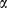 is endowed with intrinsic fitness 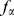 (note that the algorithm-computed fitness values are labeled with capital 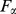). We assume that able users (those with high 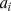) preferentially connect with high-fitness items (those with high 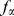). Ability and activity values are both defined in 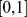 and drawn from the distribution 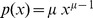 where 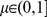 adjusts the mean value 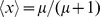 as well as the fraction of ability/activity values above 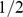 which is 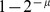.

The system evolves in discrete time steps. At each step, user 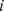 becomes active with probability 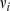. In that case:

1. With probability 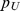, user 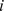 *uploads* a new item 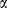 to the system. The item's fitness 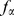 depends on the user's ability as 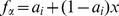, where 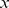 is a random variable drawn from 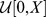. We choose this simple linear dependence of 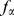 on 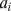 for its simplicity.

2. *Downloads* two items. The probability of choosing item 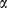 yet uncollected by user 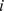 is assumed proportional to 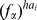 where 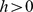.

We assume 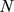 to be fixed (no new users join the community). The number of items thus grows with simulation step 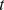 approximately as 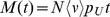 and the number of links as 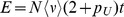. The expected network density 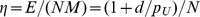 is thus constant. The number of items downloaded by an active user thus controls the final network density. If it is randomized, 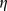 generally depends on its average value.

In our simulations, we set 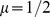 so that only 30% of users have ability/activity larger than 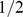. We set 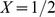 which means that despite some level of randomness, ability of a user and fitness of items submitted by them are still related. We set 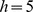 so that users with ability close to 1 are unlikely to accept items of low fitness (by contrast, users with zero ability accept items regardless of their fitness). Finally, we set 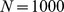 and 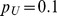 which implies network density 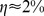 which is similar to the values seen in real systems (while density is lower for the real data that we study here, user-item networks corresponding to the classical Movielens and Netflix datasets are of a higher density [26]). We present results obtained with 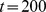 which corresponds to 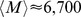 items, 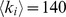, and 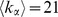. Link weights assigned to uploads and downloads are 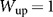 and 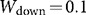 which reflects that uploading a new item is considered to be more demanding than downloading and thus deserves more reward. The influence of individual parameters on results is discussed later in this section.

To evaluate the fitness and reputation estimates obtained with the algorithm, we compute the Pearson correlation coefficient between the estimated values and their true values used in the agent-based simulation: 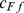 for items and 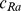 for users. To assess the bias of results towards old items and active users, we measure 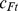 and 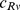, respectively. While high correlation values are desirable for the first two quantities, values close to zero are preferable for the other two.

### Model evaluation on real data

Any algorithm needs to be ultimately tested by its performance on real data. To this end, we use data obtained from the Econophysics Forum (EF, see www.unifr.ch/econophysics/) which is an online platform for interdisciplinary physics researchers and finance specialists. While there is a plenty of other online platforms where our algorithm could be applied (such as ResearchGate, Mendeley, or even arXiv), their data is not freely available and therefore we have not been able to use them for this study.

To obtain the data, we analyzed the site's weblogs created from 6th July 2010 to 31st March 2013 (1000 days in total). We removed entries created by web bots (which cause approximately 75% of the site's traffic) and all papers uploaded before 6th July 2010 (for which we do not have the full record of user actions). From all possible actions of users on the web site, we consider only interactions between users and papers uploaded to the web site. There are three distinct actions: a user can upload a paper to the site, download a paper, or view a paper's abstract. We set their respective link weights 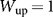, 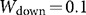, and 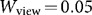 (note that 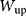 and 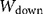 are the same as in the artificial data part). This acknowledges paper upload as the most demanding (and rare) activity and viewing an abstract signalizes paper fitness less than its direct download. Respective weights were set before evaluating the algorithms on data.

To increase the data density, we removed the users who did not upload any papers and had only one action in total. In the case of a user repeatedly interacting with a given paper, only the earliest interaction was considered. Other approaches, such as cumulating all interactions or preferring paper downloads over abstract views, for example, result in inferior performance of QR. This choice is further motivated by the fact that the first interaction does best represent the user's interest: Papers that really capture users' attention are downloaded/read immediately when encountered, whereas a later download indicates other reasons of interest. The final input data contains 5071 users, 844 papers and 29748 links, implying link density 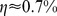. Note that the Econophysics Forum has an editor who has uploaded 85% of all papers in the analyzed sample. Paper metadata includes paper submission time, title, and a list of its authors. To avoid the problem of an author's name represented in multiple ways (*e.g.*, ‘H. Eugene Stanley’ vs ‘H. Stanley’ vs ‘HE Stanley’), we use only the first initial without comma and the surname (‘H Stanley’). As a result, there are 1527 authors in the analyzed sample. The paper metadata was augmented by citation counts, which were obtained from Google Scholar on 12th December 2013, and by the SCImago Journal Rank (SJR) of the journals where papers were eventually published. We shall use this external information to evaluate rankings of papers produced by various algorithms. We rely here on the SJR indicator instead of the perhaps more usual impact factor because the latter has been widely criticized [27]. Nevertheless, the shape of the curve presented in Fig. 3d changes little when the SJR metric is replaced with the impact factor or, for example, the journal *h*-index. The analyzed data is available in this paper's Data S1.

**Figure 2:**
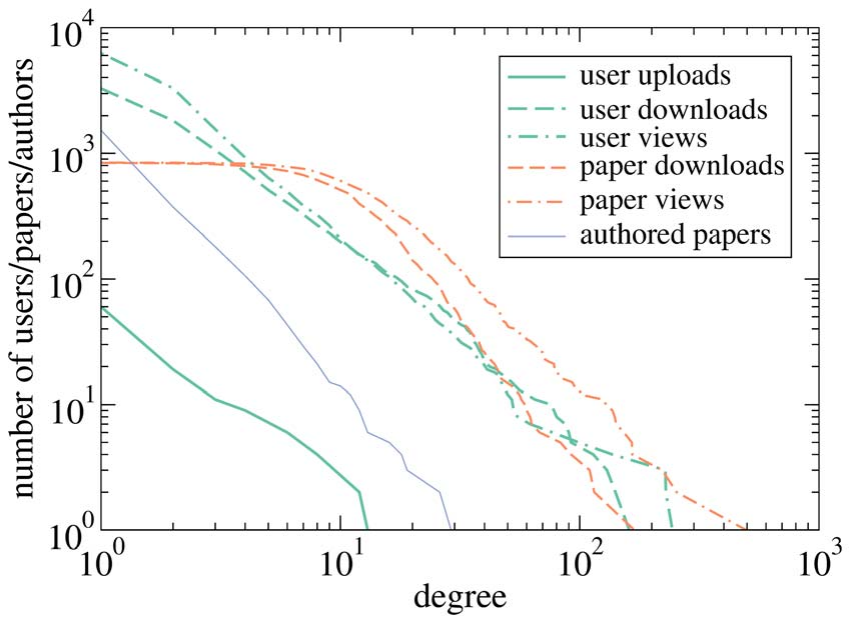
Cumulative degree distributions in the Econophysics Forum data with respect to various actions for users, papers, and authors. The editor was removed from the user upload distribution for the sake of clarity.

Figure 2 shows cumulative degree distributions for all involved parties: Users, papers, and authors. All distributions are broad and some of them might even pass statistical tests for power-law distributions. As a result, while 92% of users have ten actions in total or less, the most active users downloaded or viewed roughly a hundred of papers. With respect to the time span of the data, this is still a human level of activity which suggests that our removal of automated access was reasonably successful. The degree distribution of papers is shifted to the right as a whole with a negligible number of papers downloaded or viewed less than ten times and the most successful papers being of interest to hundreds of users. The most active authors are well-recognized in the econophysics community: Jean-Philippe Bouchaud, Shlomo Havlin, Dirk Helbing, Didier Sornette, and Eugene Stanley (in alphabetical order) have all authored more than 15 papers in the sample.

### Overview of variables

The number of users, papers, and authors are 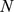, 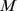, and 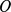, respectively. The input data is represented by a biparite network where links corresponding to paper upload, paper download, and abstract view are weighted with weights 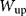, 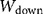, and 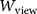, respectively. Link density in the user-paper network 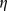 is computed as 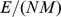 where 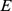 is the total number of links.

The investigated algorithms are built on vectors of item fitness 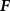, user reputation 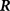 and author credit 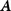. The EigenRumor algorithm's weight of author credit in an equation for item fitness is 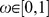, 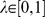 has the same function in the QRC algorithm. The QR algorithm has four parameters: 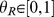 determines how to aggregate fitness of items collected by an individual user, 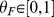 determines how to aggregate reputation of users who have collected an individual item, 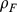 determines how much is user reputation harmed by collecting items of inferior fitness, and 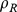 determines how much is item fitness harmed by being collected by users of inferior reputation. The QRC algorithm has the same set of parameters and three more: 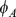 determines how to aggregate fitness of items authored by an individual author, 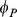 determines how to aggregate credit of authors of an individual paper, and 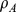 determines how much is author credit harmed by a paper of inferior fitness.

In the artificial data model, the vectors of user activity and ability are 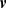 and 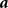, respectively. Activity and ability values of individual users are independently drawn from the distribution 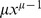 where 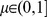 is a parameter which determines how unevenly are the values distributed (when 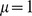, the distribution is uniform; as 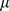 decreases, the fraction of low activity/ability users increases). The vector of item fitness is 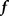. 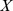 controls the correlation between item fitness and ability of the user who introduces the item in the system. 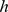 controls how selective are the users in choosing items.

## Results

### Results on artificial data

The QR algorithm has four parameters, 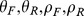, which naturally lie between 0 and 1. We evaluated the algorithm's performance for all 16 possible combinations of the limit values (0 or 1 for each of four parameters) on artificial data constructed by the model introduced above. Results for the QR setting corresponding to biHITS and two other well-performing settings, which we refer to as QR1 and QR2 from now on, are shown in Table 1.

**Table 1:** Performance of three selected parameter settings in the QR (Quality-Reputation) algorithm.

Label	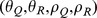	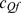	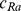	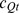	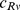
biHITS	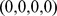	0.54	0.25	−0.58	0.93
QR1	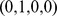	0.57	0.57	−0.57	0.15
QR2	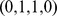	0.66	0.61	−0.46	0.02

Here 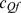, 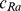, 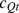 and 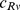 are Pearson's correlation values between estimated values 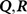 and true properties of users and papers in the agent-based model 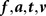.

Scores obtained with biHITS correlate least with user ability and item fitness and are at the same time biased towards old items and, even more, active users. BiHITS is therefore not a suitable algorithm for situations where item age and user activity are heterogeneous, which is often the case in real systems [28], [29]. While the problem of correlations between fitness estimates and item age is mitigated by aging which is present in most systems of this kind [30], high correlation between user activity and reputation requires additional normalization of the biHITS algorithm as done, for example, by EigenRumor or different parameterizations of QR. The well-performing variants QR1 and QR2 share two parameter values: 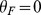 and 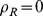. That's not surprising as the opposite values 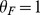 and 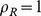 would mean that popular items are not favored over unpopular ones and that items are “punished” when users of low reputation connect with them, respectively. Settings QR1 and QR2 both achieve low correlation between reputation estimates and user activity which is due to 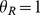 (*i.e.*, user reputation is computed as an average over user actions). The choice of 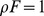 gives QR2 an advantage over QR1 in all four correlation metrics which means that it is indeed beneficial to punish users for uploading or downloading inferior content. The only quantity in which QR1 and QR2 perform badly is 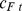 which is strongly negative for both but, as we already said, this is likely to be improved in real systems where aging of items results in eventual saturation of their degree growth.

We conclude the artificial data part with a discussion of the influence of system parameters on the presented results. The shape of user acceptance probability is determined by *h*. QR's performance improves with *h* and eventually saturates at 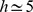. Parameters 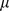 and 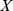 regulate the fraction of able and active users and the resulting distribution of item fitness. Our choice 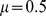 and 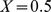 results in able/active users being a minority and the fitness distribution being rather uniform. While 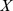 is not decisive for the algorithm's performance (though, smaller values of 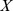 generally lead to better results), 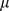 is crucial as having too few able/active users makes it impossible to detect high-fitness content. On the other hand, if able users are many, the aggregate judgment is good enough and there is no need for a sophisticated algorithm. Network sparsity 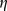 is not particularly important as long as it is not too small (then there is too little information in the system) or too large (if every item is connected to almost all users, the presence of a link loses its information value). Finally, QR results depend only on the ratio 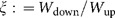 of the algorithm's parameters 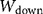 and 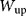. When 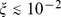, download links are of little importance and the bipartite network effectively becomes very sparse to the detriment of the QR's performance. When 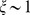, the performance deteriorates as well because upload information is almost neglected (note that there are many more downloads than uploads). Our original choice 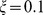 is nearly optimal.

### Results on real data

We begin our analysis by inspecting algorithms without author credit: popularity ranking (POP), where popularity is measured by the number of downloads, and bipartite HITS (biHITS). In addition, random ranking of papers (RAND) is used as the null model against which both POP and biHITS are compared. The average characteristics of top twenty papers according to these and other methods are summarized in Table 2. The expected bias towards old papers is clearly visible for the POP ranking whose top papers are on average 8 months older than RAND papers. While mean citation count of popular papers exceeds that of random papers, two of the most popular papers have never been published and four have not been cited to date: Wisdom of the crowd appears to be no good guide here. Both RAND and POP provide no information on the ranking of authors. BiHITS shows stronger bias towards old papers than POP which is probably due to its network feedback effects which reinforce its popularity-driven nature. Furthermore, it awards the Econophysics Forum editor who uploaded majority of papers with score which is so high that views and downloads by ordinary users add only small variations to the score of those papers. Even worse, papers that have not been submitted by the editor cannot reach the top of the ranking regardless of their success among the users. Thanks to normalization, the editor's weight does not represent a problem in QR1 and QR2. On the other hand, their top papers are not cited more than papers chosen by biHITS or POP. Furthermore, QR1 and QR2 choose rather popular papers and one could argue that they actually provide little new and useful information to the users. In fact, the excessive tendency of information-filtering algorithms towards popular objects is a long-standing challenge in this field [31], [32].

**Table 2:** Mean and standard error for basic metrics of top 20 papers obtained with various algorithms.

Label	Day	Down	Cit	SJR
RAND	548±41	11±1	5±1	0.5±0.1
POP	299±37	69±7	15±4	0.9±0.4
biHITS	264±34	56±7	10±3	0.7±0.2
ER	444±49	30±10	18±4	0.9±0.1
QR1	375±49	59±9	15±4	1.2±0.5
QR2	445±47	54±9	14±3	1.2±0.4
QRC	465±60	34±8	34±10	2.2±0.5

The four reported metrics are submission day (Day), number of downloads (Down), citation count (Cit), and SCImago Journal Rank (SJR) which is a measure of scientific influence of scholarly journals (an alternative to the well-known impact factor). The ER and QRC algorithm use 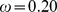 and 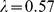, respectively.

Before analyzing ER and QRC, the parameters of QRC need to be set. We use 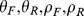 corresponding to QR1 which performed best on artificial data. We have also evaluated a variant of QRC based on QR2 and found that penalization of users connected to low fitness papers through 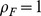 leads to negative paper scores and in turn various counter-intuitive results. To avoid assigning high credit to authors of a single successful paper (beware the trap of papers with attractive titles), we use 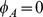 which results in accumulation of author credit over the course of time. Since 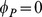 (summing the credit of a paper's authors) gives an advantage to papers with many authors, we use 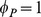. We have evaluated other possible choices of parameters 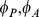 (as well as some other choices, such as paper fitness contributed by the sum of credit of two most credible authors) and found that 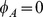 and 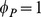 indeed produce the most satisfactory results.

**Figure 3:**
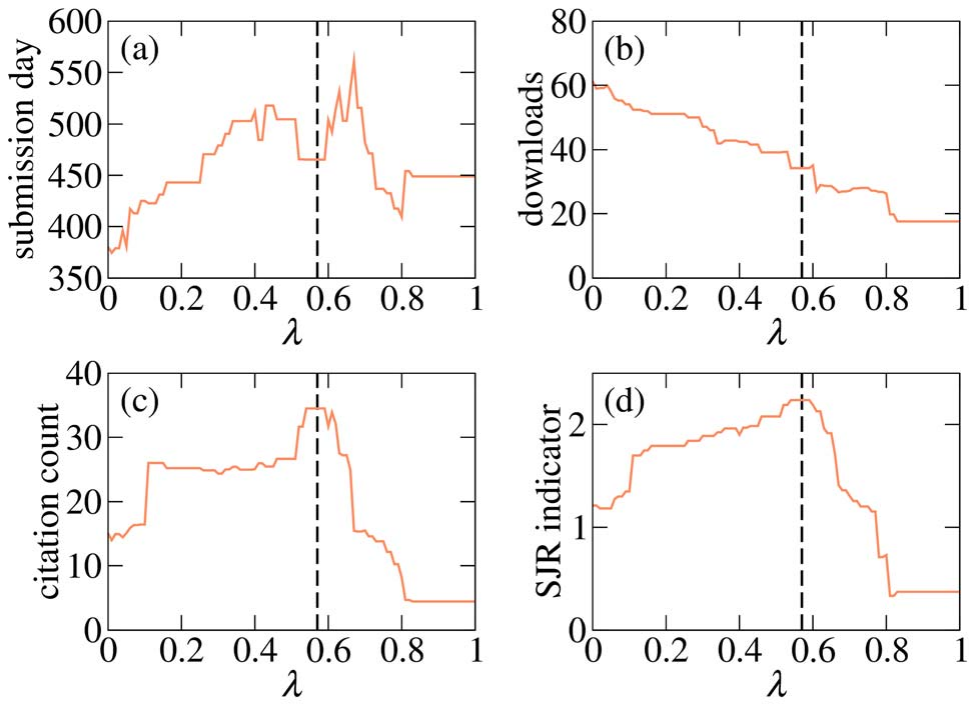
Average metrics of QRC's top 20 papers versus 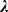. The vertical dashed line at 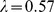 marks the setting where citation count and the SJR score are approximately maximized.

Fig. 3 shows the average metrics of the top twenty papers obtained with QRC for 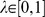. As 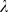 increases, the average submission day of papers in top 20 grows from 375 (the original QR1 value) to 519 when 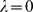; the inclusion of author credit thus helps to mitigate or even remove the time bias. The average number of downloads decreases with 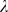 and eventually reaches less than 25% of the QR1 value. The average SJR value is improved over a wide range of 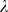 and peaks at 2.2 for 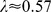. The same is true for the average citation count which peaks at 34 for 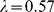. As can be seen in Table 2, QRC outperforms the other evaluated methods. The Mann-Whitney U test based on top 20 papers chosen by various algorithms confirms that QRC outperforms them at the significance level 0.02 with the exception of ER where, due to the small sample size and large fluctuations, significance is only 0.08. There are two further points to make. First, top papers chosen by QRC are generally younger than those chosen by other methods and thus have had less time to accumulate citations. Second, QRC is the only method which puts “Catastrophic Cascade of Failures in Interdependent Networks” (available on arXiv under ID 1012.0206) among the top papers. This paper with mere three citations is a summer-school version of a slightly earlier identically entitled work which has accumulated almost 500 citations (it has not been submitted to the Econophysics Forum). The paper's small contribution to the overall citation count achieved by QRC thus severely underestimates the paper's true importance. In summary, QRC's overall citation count improvement is most likely underestimated.

Since citation counts alone provide imperfect information about the fitness of scientific work, we now turn to authors. Table 3 lists top twenty authors obtained by QRC with 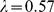 to show that they indeed include reputed names from the field of econophysics and several of their collaborators. As of December 2013, the mean *h*-index of the QRC's top 10 authors obtained by querying the Thomson's Web of Knowledge was 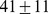 which is significantly more than 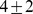 for top 12 authors (who all have identical credit) according to EigenRumor.

**Table 3:** Top 20 authors in the QRC ranking.

Rank	Name	Credit	Papers	Down
1	H. E. Stanley	0.65	26	22
2	T. Preis	0.39	8	38
3	D. Sornette	0.35	29	17
4	S. Havlin	0.22	19	11
5	B. Podobnik	0.19	8	21
6	D. Y. Kenett	0.16	11	14
7	D. Helbing	0.16	18	20
8	E. Ben-Jacob	0.14	10	12
9	A. M. Petersen	0.10	6	13
10	S. V. Buldyrev	0.09	7	13
11	J.-P. Bouchaud	0.08	16	19
12	D. Horvatic	0.07	4	20
13	B. Li	0.07	4	18
14	G. Gur-Gershgoren	0.07	5	17
15	J. J. Schneider	0.07	1	83
16	L. Feng	0.06	2	17
17	R. Woodard	0.06	6	24
18	D. Reith	0.06	1	27
19	P. Cauwels	0.06	5	12
20	A. Madi	0.06	5	11

We report here author rank, name, credit, number of authored papers (Papers), and the average number of downloads (Down). The overall average number of papers per author and downloads per paper are 1.6 and 13, respectively. The QRC algorithm uses 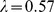.

**Figure 4:**
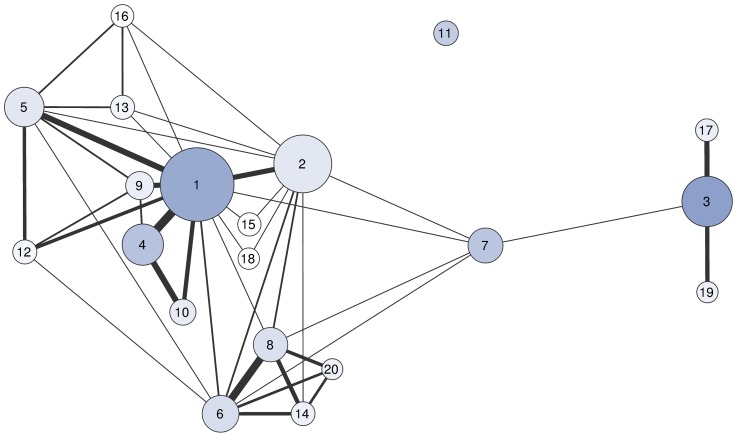
The collaboration network of 20 most-credible authors labeled with their rank in **Table 3**. Link thickness is proportional to the number of jointly authored papers. Node area is proportional to the author's credit. Node color is proportional to the number of authored papers (the darker the color, the higher the number).

**Figure 5:**
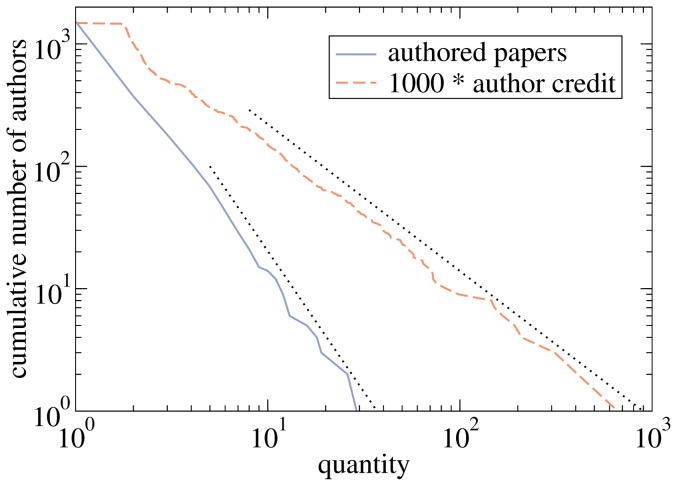
A comparison of the cumulative distribution of the number of authored papers and author credit. Dashed lines represent the results of the power-law analysis (both *α* and 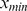).

Figure 4 visualizes the collaboration network of the QRC's top authors. This network consists of two dense communities centered around authors 1 and 6, respectively. In addition, there is author 3 with his two frequent collaborators and authors 7 and 11 whose collaboration with other top 20 authors is weak and entirely absent, respectively. Density of this network is 0.226 which is much more than the density 0.010 of the giant component of the whole author network (this giant component contains 570 nodes; the second biggest component contains 20 nodes). The high density of connections between top 20 authors is of particular importance because links within a community boost the credit of its members: high credit of one member enhances the fitness score of this member's papers which in turn enhances the credit of co-authors of these papers. Author credit in this indirect way flows between nodes of the coauthor network. The impact of mutual reinforcement of author credit can be also seen on the power-law exponent of the credit distribution which is significantly lower than the power-law exponent of the author degree distribution (see Figure 5 for a comparison of the two distributions). The standard maximum likelihood estimation and minimization of the Kolmogorov-Smirnov statistic yield the exponent 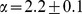 and the lower-bound of power-law behavior 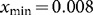 for author credit as opposed to 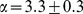 and 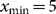 for the number of authored papers. Both fits produce high *p*-values of 0.80 and 0.90, respectively. In summary, QRC awards the most credible authors more than proportionally to the number of their papers due to mutual credit reinforcement which is mediated by the paper layer of the multilayer network.

While the overall performance of the algorithm is good, two possibly inconvenient properties can be noticed. First, the example of authors 15 and 18 shows that co-authoring one successful paper with some of the most credible authors is enough to get among the top 20 authors. Second, author 11—a highly respected figure in the field—does not collaborate with other credible authors which hinders his standing in the QRC algorithm. Both problems can be alleviated by unevenly distributing paper score among the authors with credible authors receiving higher share: This would lessen the gains of authors 15 and 18 as well as reduce gap between the most credible authors and author 11. We leave this direction for future research.

**Figure 6:**
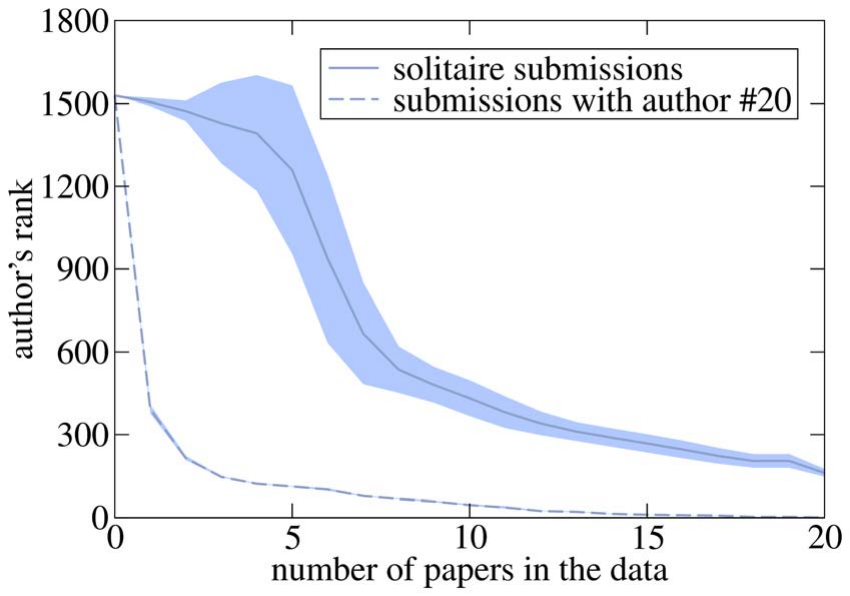
The rank of a new author gradually improves with the number of their papers in the input data. We report here a case where papers are authored only by the new author and a case where they are co-authored by author ranked 20 in Table 3. The shaded areas indicates the rank's standard deviation derived from 100 realizations).

We finally investigate the QRC's robustness with respect to a new author X who persistently submits papers of average fitness. Motivated by the previous paragraph, we consider two different scenarios: (1) X is the sole author of all papers, (2) X co-authors all papers with the last top 20 author from Table 3, A. Madi, who is assigned with substantial credit by the algorithm. We amend the real EF data by generating a certain number of papers by author X and linking each of them with 35 randomly chosen users (35 is the average paper degree in the original data) who randomly either download the paper or view its abstract; the resulting data is then used to compute X's ranking with QRC. One can see in Figure 6 that solitaire submissions result in a slow improvement of the author's rank with the number of papers. For example, this ranking is worse than 200 even after submitting 16 papers which is ten times more than the average number of papers per author in the original EF data. This slow improvement is due to the average user response to this author's papers and the absence of collaboration with other, potentially more credible authors. While the ranking improvement is much faster in the second scenario where author X co-authors all papers with a highly credible author, seven jointly authored papers are still necessary for author X to become one of top 100 authors. (Authors 15 and 18 entered top 20 after one paper thanks to collaboration with two very top authors and above-average success of their respective papers.) We can conclude that the algorithm is robust to persistent authors of low- or mediocre-fitness content.

## Discussion

We have proposed QRC, a new reputation algorithm for scientific online communities. QRC acts on a multilayer user-paper-author network and is based on three main components: *Quality* of papers, *Reputation* of users, and *Credit* of authors. We have used data from a scientific community web site, the Econophysics Forum, to evaluate the algorithm and compare its performance with that of other reputation algorithms. The newly proposed QRC algorithm outperforms those algorithms in various aspects. Papers scoring high in the resulting QRC algorithm are younger than those selected by bipartite HITS and they have been downloaded considerably fewer times than papers selected by any other algorithm considered here. At the same time, QRC's top papers have attracted significantly more citations and the SJR score of their publication venues is also higher than for papers chosen by the other algorithms. In short, QRC is able to highlight the papers that have been largely neglected by the Econophysics Forum users (as demonstrated by their relatively low number of downloads), yet they have eventually attracted considerable attention from the scientific community (as indicated by the publication venues and the citation counts). Note that QRC introduces author credit endogenously, relying on no other information than user activity on the given web site. The observed improvements are thus not achieved by providing this algorithm with more information than what is made available to the other algorithms. Furthermore, we demonstrate the presence of mutual credit reinforcement among coauthors which highlights the networking nature of the algorithms with scores propagating not only to direct network neighbors but also further down the network. We further show that QRC's top authors have on average substantially higher *h*-index than top authors found with other algorithms and that the resulting author ranking is rather robust with respect to active authors of low fitness content. The algorithm has been deployed at the Econophysics Forum where it helps to highlight valuable papers.

Our results show that the activity data from a scientific community suffices to recover a substantial part of the hierarchy of researchers in the given econophysics field. Note that the algorithm's range of applicability is not strictly limited to scientific online communities. QRC can be used in any community where: (1) shared perceptions of fitness can emerge, (2) fitness induces popularity, and (3) individual items have various authors. If a scientific community is in divide, for example, and its members deeply disagree on some theories or methods, condition (1) is violated and an attempt to produce a universal fitness ranking might be in vain. While the causality between fitness and popularity in science is imperfect (effects such as the first-mover advantage have reported [33]), it is still stronger than in music, for example, where condition (2) is questionable and the use of QRC is likely to produce dubious results. To overcome these limitations and thus extend the QRC's range of applicability remains a future challenge.

There are several research directions which remain open. The behavior and performance of the QRC algorithm upon non-integer choices of its parameters (such as the exponent 0.5 used in Eq. (6)) need to be examined. However, to obtain statistically robust results, additional datasets need to be obtained before attempting this kind of high-dimensional optimization task. User surveys can be employed as an additional evaluation tool complementing the current quantitative approach based on citations, journal quality measured by the SJR score and *h*-index. Notably, the QRC algorithm has been deployed at the Econophysics Forum which provides an opportunity to study the algorithm's impact on the users' behavior and the web site's usage. The aforementioned possibility of non-uniform distribution of paper score among the paper's authors might award more long-term leaders with many successful papers. Study of other forms of gaming and spamming of the algorithm is necessary in order to understand its limits of robustness. While co-authorship information impacts the author credit in QRC (see the difference between solitaire submissions and submissions with author #20 in Fig. 6), one might also consider making the co-authorship contribution explicit as in the previous QTR algorithm. For input data exceeding the three-year time span of the presently studied Econophysics Forum data, it may be suitable to introduce time decay of fitness and credit values to prevent the oldest contributions and the most active authors from occupying top positions in their respective rankings. Results presented in [24], [30] may provide a starting ground for these efforts. One should not forget that the QRC results are community-specific as they are based on feedback of a given group of users. This is not only a limitation but also an opportunity: The QRC algorithm can be eventually used to study the dynamics and differences between various research communities.

## Supporting Information

Data S1 **Datasets.**
(ZIP)

## References

[1] Radicchi F, Fortunato S, Castellano C (2008) Universality of citation distributions: Toward an objective measure of scientific impact. Proceedings of the National Academy of Sciences 105:17268–17272. 10.1073/pnas.0806977105 PMC2582263 18978030

[2] Larivière V, Archambault É, Gingras Y (2008) Long-term variations in the aging of scientific literature: From exponential growth to steady-state science (1900–2004). Journal of the American Society for Information Science and technology 59:288–296.

[3] Maxmen A (2010) Science networking gets serious. Cell 141:387–389. 20434976 10.1016/j.cell.2010.04.019

[4] Resnick P, Kuwabara K, Zeckhauser R, Friedman E (2000) Reputation systems. Communications of the ACM 43:45–48.

[5] Sabater J, Sierra C (2005) Review on computational trust and reputation models. Artificial intelligence review 24:33–60.

[6] Axelrod RM (2006) The evolution of cooperation. Basic books.

[7] Masum H, Zhang YC (2004) Manifesto for the reputation society. First Monday 9.

[8] Jøsang A, Ismail R, Boyd C (2007) A survey of trust and reputation systems for online service provision. Decision support systems 43:618–644.

[9] Hanrahan BV, Convertino G, Nelson L (2012) Modeling problem difficulty and expertise in stack-overflow. In: Proceedings of the ACM 2012 conference on Computer Supported Cooperative Work Companion. ACM, pp.91–94.

[10] Newman M (2010) Networks: an introduction. Oxford University Press.

[11] Brin S, Page L (1998) The anatomy of a large-scale hypertextual web search engine. Computer networks and ISDN systems 30:107–117.

[12] Franceschet M (2011) Pagerank: Standing on the shoulders of giants. Communications of the ACM 54:92–101.

[13] Kleinberg JM (1999) Authoritative sources in a hyperlinked environment. Journal of the ACM (JACM) 46:604–632.

[14] Fujimura K, Tanimoto N (2005) The eigenrumor algorithm for calculating contributions in cyberspace communities. In: Trusting Agents for Trusting Electronic Societies, Springer. pp.59–74.

[15] Deng H, Lyu MR, King I (2009) A generalized co-hits algorithm and its application to bipartite graphs. In: Proceedings of the 15th ACM SIGKDD international conference on Knowledge discovery and data mining. ACM, pp.239–248.

[16] Liao H, Cimini G, Medo M (2012) Measuring quality, reputation and trust in online communities. In: Foundations of Intelligent Systems, Springer. pp.405–414.

[17] Chen P, Xie H, Maslov S, Redner S (2007) Finding scientific gems with googles pagerank algorithm. Journal of Informetrics 1:8–15.

[18] Radicchi F, Fortunato S, Markines B, Vespignani A (2009) Diffusion of scientific credits and the ranking of scientists. Physical Review E 80:056103. 10.1103/PhysRevE.80.056103 20365039

[19] Zhou YB, Lü L, Li M (2012) Quantifying the influence of scientists and their publications: distinguishing between prestige and popularity. New Journal of Physics 14:033033.

[20] De Domenico M, Solé-Ribalta A, Cozzo E, Kivelä M, Moreno Y, et al (2013) Mathematical formulation of multilayer networks. Physical Review X 3:041022.

[21] Castillo C, Donato D, Gionis A (2007) Estimating number of citations using author reputation. In: String processing and information retrieval. Springer, pp.107–117.

[22] Acuna DE, Allesina S, Kording KP (2012) Future impact: Predicting scientific success. Nature 489:201–202. 22972278 10.1038/489201a PMC3770471

[23] Petersen AM, Stanley HE, Succi S (2011) Statistical regularities in the rank-citation profile of scientists. Scientific reports 1. 10.1038/srep00181 PMC3240955 22355696

[24] Wang D, Song C, Barabási AL (2013) Quantifying long-term scientific impact. Science 342:127–132. 24092745 10.1126/science.1237825

[25] Gilbert N (2008) Agent-based models. Sage.

[26] Lü L, Medo M, Yeung CH, Zhang YC, Zhang ZK, et al (2012) Recommender systems. Physics Reports 519:1–49.

[27] Brembs B, Button K, Munafò M (2013) Deep impact: unintended consequences of journal rank. Frontiers in human Neuroscience 7. 10.3389/fnhum.2013.00291 PMC3690355 23805088

[28] Vázquez A, Oliveira JG, Dezsö Z, Goh KI, Kondor I, et al (2006) Modeling bursts and heavy tails in human dynamics. Physical Review E 73:036127. 10.1103/PhysRevE.73.036127 16605618

[29] Zhou T, Medo M, Cimini G, Zhang ZK, Zhang YC (2011) Emergence of scale-free leadership structure in social recommender systems. PLoS One 6:e20648. 21857891 10.1371/journal.pone.0020648 PMC3152579

[30] Medo M, Cimini G, Gualdi S (2011) Temporal effects in the growth of networks. Physical review letters 107:238701. 22182132 10.1103/PhysRevLett.107.238701

[31] Adomavicius G, Kwon Y (2012) Improving aggregate recommendation diversity using ranking-based techniques. Knowledge and Data Engineering, IEEE Transactions on 24:896–911.

[32] Gualdi S, Medo M, Zhang YC (2013) Crowd avoidance and diversity in socio-economic systems and recommendations. EPL (Europhysics Letters) 101:20008.

[33] Newman M (2009) The first-mover advantage in scientific publication. EPL (Europhysics Letters) 86:68001.

